# Justification of Genetic Factors for Predicting the Risk of Acute Bleeding in Peptic Ulcer Disease

**DOI:** 10.25122/jml-2020-0041

**Published:** 2020

**Authors:** Fedir Vasilyevich Grynchuk, Ivan Ivanovich Dutka, Iryna Ihorivna Panchuk, Roman Anatolyevich Volkov, Michael Ivanovich Sheremet, Vitaliy Vasilyevich Maksymyuk, Volodymyr Volodymyrovich Tarabanchuk, Ihor Ivanovich Bilyk, Yuriy Mykolayovych Myshkovskii

**Affiliations:** 1. First Department of Surgery, Higher State Educational Establishment “Bukovinian State Medical University”, Chernivtsi, Ukraine; 2. Department of Molecular Genetics and Biotechnology, Yuriy Fedkovych Chernivtsi National University, Institute of Biology, Chemistry and Bioresources, Chernivtsi, Ukraine; 3. Department of General Surgery, Higher State Educational Establishment “Bukovinian State Medical University”, Chernivtsi, Ukraine

**Keywords:** gene PAI-1 (SERPINE 1), peptic ulcer disease, ulcer bleeding, recurrent ulcerative bleeding, prediction, predictors of recurrent ulcerative bleeding

## Abstract

PAI genotyping for the G43A and 4G/5G polymorphisms was performed in 60 patients with peptic ulcer disease: 12 with an uncomplicated ulcer, 5 with perforation, the rest with ongoing bleeding. Fourteen patients had recurrent bleeding. The 5G/5G and G43A genotypes were not detected in patients with uncomplicated ulcers. All patients with ulcer perforation had the G43G genotype, 60% of patients had the 4G/4G genotype, and the rest of them had the 4G/5G and 5G/5G genotypes. The number of carriers of the 5G allele (86.05%) was higher in patients with bleeding than in ones with ulcer perforation (p=0.036) and ulcer without bleeding (p=0.021, χ2=5.32). The number of carriers of the 5G allele was higher in patients with recurrent bleeding (92.86%) than those without any relapses (82.76%) but there were no statistically significant differences (p=0.27, χ2=0.802). The G43G homozygous genotype was found in 94.12% of patients with peptic ulcer without bleeding, which was statistically significantly higher (p=0.02) than the ones with bleeding. The A allele was observed in 27.91% of patients with bleeding and 8.33% patients without any bleeding (p=0.05). The number of carriers of the A allele in patients with recurrent bleeding was statistically significantly higher than in ones without any bleeding (p=0.046). The 5G and A alleles in patients with a peptic ulcer can be used to predict the course of peptic ulcer disease and can be regarded as a predictor of the risk of bleeding relapse.

## Introduction

Peptic ulcer disease is an urgent medical problem [1-5]. Peptic ulcer bleedings are the main cause of non-variceal upper gastrointestinal bleeding [2-5]. The morbidity in patients with bleeding peptic ulcers reaches 8-10% and increases when recurrent bleeding occurs [5, 6]. The imperfection of prognostic scales is one of the reasons for high morbidity. The most common scales are Rockall, GlasgowBlatchford, Baylor, Cedars-Sinai, AIMS65, PNED [7-10]. However, according to some authors, more accurate scales are needed [8], and this is why they suggest using additional predictive criteria [8, 11, 12]. A common flaw of the known scales is that they are based exceptionally on clinical criteria and do not take into account the mechanisms of bleeding development. One of these mechanisms is excessive activation of fibrinolysis and inhibition of anti-fibrinolytic factors, which, in particular, is proved in our studies [13], even though the reasons are not known precisely. At the same time, mutations of the PAI-1 (Plasminogen activator inhibitor- 1) gene (SERPINE 1) are known to cause bleedings. This gene encodes the PAI-1 protein, an inhibitor of the plasminogen activator, which is a crucial regulator of the fibrinolytic system [14, 15]. PAI-1 is the main inhibitor of tissue plasminogen activator (tPA) and urokinase (uPA) [16]. These two proteins are the main activators of plasminogen, which convert plasminogen into plasmin [17].

In the case of congenital PAI-1 deficiency, the occurrence of hemorrhagic diathesis and increased tissue bleeding can be found in injured patients [15, 18]. 4G/5G polymorphism in the РАІ-1 gene promoter may be a risk factor for recurrent ulcer bleeding [19]. However, the clinical significance of other variants of РАІ-1 gene polymorphism has not been studied yet, although it has been noted that this can cause various disorders of thrombosis, regeneration, and others [14, 16, 20].

## Material and Methods

The study involved 60 patients with peptic ulcer disease. Forty-two (70%) were men and 18 (30%) women aged from 21 to 83, and the average age was 52.08±2.12 years. All patients have been the residents of the Chernivtsi region of Ukraine and belong to the white race. Thirty-seven (61.67%) patients have had a duodenal ulcer, and the rest (38.33%) a gastric ulcer. Twelve of them have had an uncomplicated ulcer, 3 (27.27%) females and 8 (72.73%) males and the average age was 46.91±4.04 years. Five (8.33%) patients have had a perforated ulcer, all males and the average age has been 35.78±3.48 years. Forty-three (71.67%) patients had ulcers complicated by acute bleeding, and 14 (32.56%) were females, 29 (67.44%) males, the average age being 55.72±1.81 years. In 29 (67.44%) patients, the bleeding has been stopped conservatively. Eleven of them (18.33%) have had an ulcer for the first time, 9 (15%) of the patients had a history of ulcer, and 9 a history of bleeding ulcer. Fourteen (32.56%) patients have had recurrent bleeding; of them, 4 (28.57) were females, 10 (71.43%) males, and the average age was 57.41±3.04 years. There was no significant difference in demographic indicators among patients. Half of the patients have had their bleeding stopped by injecting hemostatic agents, and the rest were subject to an operative treatment.

None of the patients took nonsteroidal anti-inflammatory drugs one year before the hospital admission. There were 5 (36.36%) smokers among patients with uncomplicated ulcers, 2 (40%) smokers among patients with ulcer perforation, 16 (37.21%) smokers among patients with ulcerative bleeding, and 5 (35.71%) smokers among patients with recurrent bleeding. With the help of the rapid urease test, H. pylori was found in 8 (72.73%) patients with uncomplicated ulcer, in 4 (80%) patients with ulcer perforation, in 33 (76.74%) patients with ulcerative bleeding, and 11 (78.57%) patients with recurrent bleeding. So there was no significant difference in these indicators among patients.

PAI genotyping by G43A and 4G/5G polymorphism was performed by using polymerase chain reaction (PCR). To be able to do this, a standard genomic DNA was extracted from the blood of the probands, using a set of reagents to isolate DNA from the clinical material (“DNA-Sorb B”). The PCR amplification of the corresponding section of the PAI gene was performed using a specific pair of primers 5’ - CAC AGA GAG AGT CTG GCC ACG -3’ and 5’ - CCA ACA GAG GAC TCT TGG TC -3’, according to Jeon et al. [21]. The amount of DNA for PCR was 50 ng per reaction. The amplification reaction mix also contained 1^x^ standard Maxima Hot Start Green PCR Master Mix (Thermo Scientific, USA) and primers of 1 mm each. The total volume of the reaction mixture was 50 μl. PCR was performed using the CFX96 amplifier (Bio-Rad, USA) with the help of the following program:

1. Initial activation of DNA polymerase-95°C, 4 min
2. Denaturation of DNA-94°C, 45 sec
3. Hybridization of primers-60°C, 30 sec
4. DNA synthesis-72°C, 1 min
5. Termination of amplification-72°C, 8 min
6. Termination of the reaction –4°C.

The total number of amplification cycles was 35. PCR results were analyzed by electrophoresis in 2% agarose gel using a Tris-borate buffer [14]. To visualize the DNA fragments, the gel was stained with ethidium bromide and photographed in ultraviolet light on the GelDoc 2000 set (BioRad, USA). Their electrophoretic mobility was compared with the mobility of the DNA marker Gene Ruler DNA Leader Mix (Thermo Scientific) to determine the length of the obtained fragments. The length of the resulting product for the 4G/5G polymorphism was 99 PN and 266 NN for the G43A polymorphism, which was expected. The received PCR products have been processed with the BoxI (Thermo Scientific) restriction enzyme. The processing of the PCR product was conducted with the restriction enzyme and according to the recommendations of the enzyme manufacturer (Thermo Scientific). The obtained fragments processed with the restriction enzyme were analyzed by electrophoresis in 4% agarose gel.

The statistical dependence between the values was checked by determining the Fisher criterion, the Pearson’s χ2 criterion, in particular, the correspondence of the genotype distribution to the hardy-Weinberg equilibrium. The critical significance level for testing statistical hypotheses in this study was assumed to be 0.05. The analysis was performed using Microsoft® Office Excel tables (build 11.5612.5703).

## Results and Discussion

In the genotyping of the 4G/5G polymorphism of the PAI-1 gene in the examined patients, the following genotype distribution was considered: 4G/4G – 13 homozygotes, 5g/5G – 13 homozygotes, and 4G/5G – 34 heterozygotes. The frequency of both alleles, 4G and 5G, is 0.5. The relevant heterozygosity is Но=0.57, while the theoretical (expected) heterozygosity is Не=0,50. Genotyping of the G43A polymorphism of the PAI-1 gene in the examined patients has established the following distribution by genotype: GG, homozygote by wild-type allele-45, GA, heterozygote11 and AA, homozygote by mutant alleles-2 people. The frequency of the G allele was 0.870, and that of the A allele was 0.130. The relevant heterozygosity is Но=0.19, while the theoretical (expected) heterozygosity is Не=0.226.

None of the patients with uncomplicated ulcers have had the 5G/5G homozygous genotype. At the same time, this kind of genotype was found in 12 (27.91%) patients with bleeding (p=0.027). In general, the 5G allele has occurred in most patients with bleeding peptic ulcers (n=37, 86.05%) (Table 1).

**Table 1:** Variants of the 5G4 polymorphism of the PAI-1 gene in the examined patients.

**No.**	**Group**	**4G/4G, % (n)**	**5G/5G+5G/4G, % (n)**
**1**	Without acute complications, n=12	33.33 (4)	66.67 (8)
**2**	With perforation, n=5	60 (3)	40 (2)
**3**	Without bleeding, total n=17	41.18 (7)	58.82 (10)
**4**	With bleeding, total n=43	13.95 (6)	86.05 (37), p2-4=0.036, p3-4=0.021 χ^2^=5.32
**5**	Without recurrence of Bleeding, n=29	17.24 (5)	82.76 (24)
**6**	With recurrent bleeding, n=14	7.14 (1)	92.86 (13), Р2-6=0.036, Р3-6=0.034
**7**	With a history of bleeding, n=9	0	100 (9)
**8**	With the first detected bleeding only, n=34	35.29 (12)	64.71 (22), Р7-8=0.036

The 5G allele carriers (genotypes 5G/5G and 4G/5G) were significantly higher among patients with bleeding peptic ulcers rather than ones with ulcer perforation (p=0.036) and ulcer without bleeding (p=0,021, χ2=5.32). We found a significantly higher level of 5G allele carriers among patients with recurrent bleeding than those with ulcer perforation (p=0.036) and ulcers without bleeding (Р=0,034). The 5G allele was seen more often in patients with recurrent bleeding (92.86%) than in ones without relapses (82.76%), but there were no statistically significant differences (p=0.27, χ2=0.802). There was no patient with the 4G/4G homozygous genotype among patients with a history of bleeding, and this is found statistically less frequently than in patients with first-time bleeding (p=0.036).

None of the patients with uncomplicated and perforated ulcers had the G43A heterozygous genotype. One patient with an uncomplicated ulcer had the A43A homozygous genotype. In general, the G43G homozygous genotype was found in 94.12% of patients without bleeding. This is statistically significantly more frequent (p=0.02) than in patients with bleeding, among whom the homozygous genotype G43G was found in 72.09% cases and the heterozygous genotype G43A in 36.36%.

The A allele was observed in 12 (27.91%) patients with bleeding and one (8.33%) patient without any bleeding (p=0.05) (Table 2). The number of carriers of the A allele in patients with recurrent bleeding has been statistically significantly higher than in patients without any bleeding (p=0.046). There has been no one with the A allele among patients with a history of bleeding. This is statistically significantly less than in patients with first-time bleeding (p=0.036).

**Table 2:** Variants of the G43A polymorphism of the PAI-1 gene in the examined patients.

**No.**	**Group**	**G43G, % (n)**	**G43A+A43A, % (n)**
**1**	Without acute complications, n=12	91.67 (11)	8.33 (1)
**2**	Perforation of the ulcer. n=5	100 (5)	0
**3**	Without bleeding, total n=17	94.12 (16)	5.88 (1)
**4**	With bleeding, total n=43	72.09 (31)	27.91 (12), p3-4=0.05
**5**	Without recurrence of Bleeding, n=29	75.86 (22)	24.14 (7)
**6**	With recurrent bleeding, n=14	64.29 (9)	35.71 (5), p3-6=0.046
**7**	With a history of bleeding, n=9	100 (9)	0
**8**	With the first detected bleeding only, n=34	64.71 (22)	35.29 (12), p7-8=0.036

Therefore, the conducted analysis indicated that the 5G and A alleles of the PAI-1 gene in the examined patients are associated with the occurrence of acute ulcerous bleeding. This research supports the statement about the role of mutations of the PAI-1 gene, which encodes an inhibitor of the plasminogen activator 1 in the occurrence of ulcerative bleeding [19].

The results show that taking into account variants of the PAI-1 gene genotype can be used to predict the course of peptic ulcer disease. The 5G and A alleles of the PAI-1 gene in patients with acute ulcerous bleeding could be considered a predictor of the risk of recurrence of bleeding.

## Conclusions

The number of carriers of a homozygous genotype (4G/4G) in the 4G/5G polymorphism of PAI-1 is 33.33% among the examined patients with uncomplicated peptic ulcer disease. The rest of them are carriers of the 4G/5G genotype. The number of carriers of the homozygous GG genotype in the G43A polymorphism of PAI-1 is 91.67%, and the rest are carriers of the AA genotype. No homozygote genotype (5G/5G) or heterozygous genotype (G43А) were detected in any of the cases. 

All patients with ulcer perforation have had the G43G genotype, 60% of patients had the 4G/4G genotype, and the rest had the 4G/5G and 5G/5G genotypes.

The number of carriers of the 5G allele (86.05%) was statistically significantly higher in patients with peptic ulcer bleeding than in ones with ulcer perforation (p=0.036) and peptic ulcer without bleeding (p<0.01, χ2=5.32); the number of carriers of the 5G allele was also higher in patients with recurrent bleeding (92.86%) than in ones without recurrent bleeding (82.76%), though without any statistically significant differences (p=0.27, χ2=0.802).

The G43G homozygous genotype was found in 94.12% of patients with peptic ulcer without bleeding, which was statistically significantly higher (p=0.02) than in patients with bleeding.

The number of carriers of the A allele was higher among patients with bleeding from a peptic ulcer (27.91%) than in ones without bleeding (8.33%), though without any statistically significant differences (p=0.05); at the same time, the number of carriers of the A allele in patients with recurrent bleeding has been statistically significantly higher than in ones with peptic ulcer without bleeding (p=0.046).

The 5G and A alleles of the PAI-1 gene in the examined patients are associated with the occurrence of bleeding from a peptic ulcer.

The variants of the PAI-1 gene genotype could be used to predict the course of peptic ulcer disease, and the 5G and A alleles of the PAI-1 gene in patients with bleeding from a peptic ulcer could be considered a predictor of the risk of recurrence of bleeding.

## Conflict of Interest

The authors declare that there is no conflict of interest.
